# Heightened inflammasome activation is linked to age-related cognitive impairment in Fischer 344 rats

**DOI:** 10.1186/1471-2202-12-123

**Published:** 2011-12-01

**Authors:** Lana J Mawhinney, Juan Pablo de Rivero Vaccari, Gordon A Dale, Robert W Keane, Helen M Bramlett

**Affiliations:** 1 Department of Neurological Surgery, University of Miami Miller School of Medicine, 1095 NW 14th Terrace, Miami, FL 33136, USA; 2 The Miami Project to Cure Paralysis, University of Miami Miller School of Medicine, 1095 NW 14th Terrace, Miami, FL 33136, USA; 3 Department of Physiology and Biophysics, University of Miami Miller School of Medicine, 1600 NW 10th Avenue, Miami, Florida 33136, USA; 4 Bruce W. Carter Department of Veterans Affairs Medical Center, 1201 N.W. 16th Street Miami, FL 33125, USA

## Abstract

**Background:**

Members of the mammalian nucleotide binding domain, leucine-rich repeat (LRR)-containing receptor (NLR) family of proteins are key modulators of innate immunity regulating inflammation. Our previous work has shown that among the members of this family, NLRP1/NALP1, present in neurons, plays a crucial role in inflammasome formation and the production of the inflammatory cytokines interleukin (IL) -1β and IL-18 after various types of central nervous system injury.

**Results:**

We investigated whether age-related cognitive decline may involve a heightened inflammatory response associated with activation of the NLRP1 inflammasome in the hippocampus. Young (3 months) and aged (18 months) male Fischer 344 rats were tested in a spatial acquisition task via Morris water maze. Following behavioral testing, hippocampal lysates were assayed for expression of NLRP1 inflammasome components and inflammatory cytokines. Hippocampal lysates from aged rats showed significantly higher levels of NLRP1 inflammasome constituents, caspase-1, caspase-11, the purinergic receptor P2X7, pannexin-1 and X-linked inhibitor of apoptosis (XIAP) than lysates from younger animals. Following treatment with probenecid, an inhibitor or pannexin-1, aged animals demonstrated reduction in inflammasome activation and improvement in spatial learning performance.

**Conclusions:**

Our behavioral findings are consistent with increases in IL-1β and IL-18 that have been previously shown to correlate with spatial learning deficits. Probenecid reduced activated caspase-1 and ameliorated spatial learning deficits in aged rats. Thus, aging processes stimulate activation of the NLRP1 inflammasome and secretion of IL-1β and IL-18 that may contribute to age-related cognitive decline in the growing elderly population. Moreover, probenecid may be potentially useful as a therapy to improve cognitive outcomes in the aging population.

## Background

Inflammatory immune responses are induced in the immune privileged central nervous system (CNS) by aging and neurodegenerative diseases, but CNS cells and molecular mechanisms that regulate innate immunity are poorly defined. One prominent and early inflammatory cytokine present in aged and diseased tissue is IL-1β that may contribute to secondary cell death. Despite the central function attributed to the immune system in aging and neurodegenerative diseases, the initiating signaling pathways that ultimately lead to the activation of the CNS innate immune system are unclear.

The inflammatory cytokine IL-1β is normally produced and stored in the cytosol as inactive pro-IL-β that is rapidly cleaved by caspase-1, which then elicits inflammation after being released from the cell. A number of host-derived molecules that alert the innate immune system to cell injury and tissue damage activate the inflammasome [1]. These molecules called "danger-associated molecular patterns" include ATP [2] and high extracellular potassium [3]. Our recent work has shown that the NLRP1 inflammasome in neurons plays a crucial role in the innate CNS immune response induced by injury [3-6], but whether the NLRP1 inflammasome contributes to inflammatory cytokine production and cognitive impairment due to aging remains largely undefined.

Amplified and prolonged inflammatory responses in the aged brain have been reported to promote cognitive and behavioral impairments [7,8], and clinical studies of elderly patients with infections reveal increases in the occurrence of delirium [9,10]. Moreover, elderly patients with prolonged infections are also prone to an increased probability of developing dementia [11] and increased mortality rate [12]. Thus, the prolonged, amplified production of inflammatory molecules may underlie a heightened neuroinflammatory response in the aged brain leading to cognitive impairments.

In the present study, we found that the NLRP1 inflammasome is involved in age-induced activation of caspase-1, and the release of mature IL-1β and IL-18 in the hippocampus. Moreover, aging induced increased expression and altered cellular distribution of critical components of the NLRP1 inflammasome in hippocampal neurons. These changes corresponded to age-related cognitive deficits in spatial learning. Treatment with probenecid, an anti-inflammatory drug commonly used for gout, reduced NLRP1 inflammasome activation and improved spatial learning performance in aged rats. Thus, it appears that heightened NLRP1 inflammasome activity is induced by the natural aging process resulting in increased inflammatory cytokine production and cognitive impairment that can be attenuated by inhibition of inflammasome activation.

## Results

### Aged animals perform poorly on a hippocampal-dependent spatial learning task

**Figure 1:**
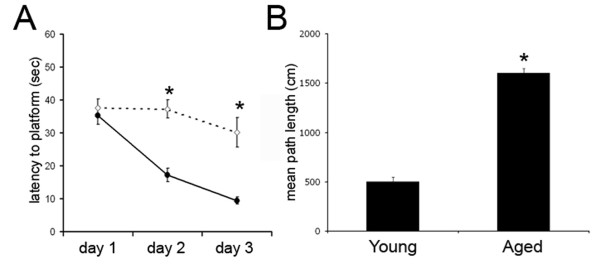
**Aged animals perform poorly on a hippocampal dependent spatial learning task**. Average latency to platform on day 1-3 of spatial acquisition testing via Morris water maze for young (closed circles) and aged rats (open circles) (**A**). Mean path length for young and aged rats on final day of testing (**B**). Data are presented as mean +/- SEM. *p < 0.01 compared with young. n = 20 per group.

To assess age-related deficits in hippocampal-dependent spatial learning, young and aged rats were tested in a 3-day spatial acquisition task via Morris water maze (Figure 1). Mean (+/- standard error mean (SEM)) values were determined for each testing day on latency to platform (Figure 1A) and for testing day 3 for mean path length (Figure 1B) For days 2 and 3, young rats located the hidden platform with lower latencies (day 2: 17.1 +/- 1.4 s, day 3: 9.4 +/- 0.7 s) than aged rats (day 2: 35.8+/- 1.3 s, day 3: 29.3 +/- 1.4 s) (Figure 1A). Additionally, mean path-length for young rats (502 +/- 48 cm) was significantly shorter than for aged rats (1487 +/- 132 cm) on day 3 of testing. Aged rats are consistently impaired in learning the location of the escape platform [13]. Therefore, our results were similar to previous findings that aged rats demonstrate spatial learning impairment compared to young rats.

### Aging increases hippocampal expression of NLRP1 inflammasome components in the hippocampus

**Figure 2:**
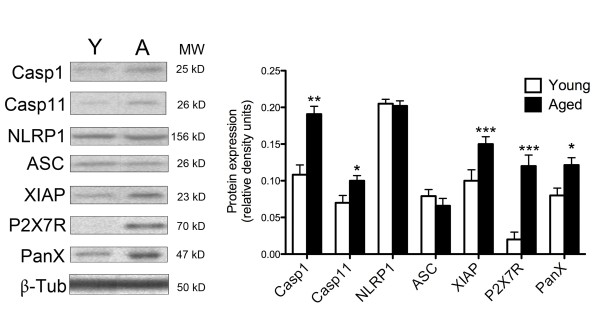
**Aging alters expression of NLRP1 inflammasome components**. Representative immunoblots and densitometric analysis of immunoblots for caspase-1 (Casp1), caspase-11 (Casp11), NLRP1, ASC, cleaved XIAP, P2X7 receptor (P2X7R) and pannexin-1 (PanX1) in brain lysates of young (Y) and aged (A) animals. Protein levels of cleaved caspase-1 and -11 are higher in aged animals compared to young. Protein levels of NLRP1 and ASC did not change with age whereas P2X7 receptor, the pannexin-1 protein and the cleaved fragment of XIAP are higher in aged animals than in young animals. β-Tubulin was used as an internal standard and control for protein loading. Data are presented as mean +/- SEM. *p < 0.05, **p < 0.01, ***p < 0.005 compared with young. n = 6 per group.

The NLRP1 inflammasome in neurons constitutes a multiprotein complex consisting of caspase-1, caspase-11, apoptosis-associated-speck-like protein containing a caspase recruitment domain (ASC), NLRP1, P2X7, pannexin-1 and XIAP [4]. Upon inflammasome activation caspase-1 is cleaved into active fragments that process pro-IL-1β and pro-IL-18 into active IL-1β and IL-18 [14]. To determine whether aging alters expression of NLRP1 inflammasome components, protein lysates of young and aged hippocampi were analyzed for levels of inflammatory caspases and key inflammasome proteins (Figure 2). Hippocampal lysates from aged animals contained significantly higher levels of cleaved fragments of caspase-1 (p = 0.006), caspase-11 (p = 0.011), and XIAP (p = 0.0021), and increased levels of the P2X7 receptor (p = 0.001) and pannexin-1 (p = 0.049) than lysates from young rats. Levels of NLRP1 and ASC were not altered in the aged animals. Thus, aging stimulates the expression of some of the key components of the NLRP1 inflammasome in the hippocampus, suggesting an involvement of the inflammasome in the aging process.

### Aging induces alterations in expression patterns of pannexin-1 and the purinergic receptor P2X7 in the hippocampus

**Figure 3:**
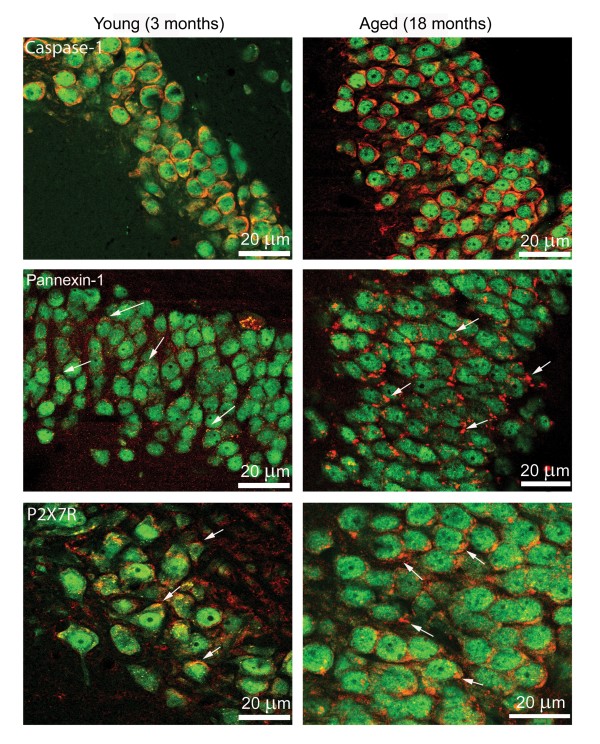
**Aging induces alterations in protein expression patterns of Caspase-1, pannexin-1 and P2X7R in hippocampal neurons**. Confocal images show hippocampal neurons in the CA3 region of young and aged animals. Sections were stained for caspase-1, pannexin-1 and P2X7 (red) and the neuronal marker NeuN (green). In aged animals, the immunoreactivity of caspase-1, P2X7 and pannexin-1 are increased in neurons of the hippocampus compared to young animals with P2X7 and pannexin-1 showing a polarized distribution near the somatic membrane (arrows). Bar=20 μm.

In our previous work, we showed that the pore-forming protein pannexin-1 can transport extracellular NLRP1 stimuli into the cytoplasm of neurons and astrocytes after P2X7 receptors bind ATP [3], suggesting that K^+ ^ion influx through pannexin-1 pores may initiate activation of the NLRP1 inflammasome. To establish whether aging induces changes in the pattern of expression of caspase-1, pannexin-1 and the P2X7 receptor, hippocampi from young (n = 4) and aged rats (n = 6) were examined by confocal microscopy. Brain sections underwent immunohistochemical procedures followed by confocal microscopy. Images indicate that aging results in altered staining patterns of inflammasome proteins in the hippocampus (Figure 3). In young rats, caspase-1 immunoreactivity (red) was present in NeuN positive nuclei (green) in a diffuse expression pattern, while intense caspase-1 staining was seen in hippocampal neurons of aged rats located in the cell nucleus and cytoplasm. The increased intensity of caspase-1 staining was consistent across aged animals. A more striking alteration was observed in the immunostaining of pannexin-1 and P2X7 receptors. In aged rats, immunoreactivity of both inflammasome proteins was markedly enhanced, and intense patchy staining was seen in the neuronal soma near or associated with the plasma membrane (arrows). The intensity of this patchy staining was greater in the apical portion of the hippocampal neuronal membrane (arrows) than in the basal portion. The staining patterns of aged animals were more variable than young animals. However, the appearance of intense patches of pannexin-1 and P2X7 receptors was present in the hippocampus of all six aged animals analyzed. Labeling patterns were similar throughout the hippocampus for young and aged rats. These results demonstrate that aging induces dramatic alterations in the expression patterns of caspase-1, pannexin-1 and P2X7 receptors in hippocampal neurons.

### Probenecid treatment attenuates age-related elevation in NLRP1 inflammasome components

**Figure 4:**
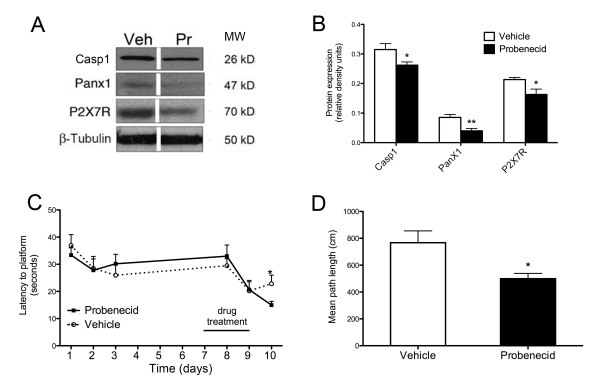
**Probenecid reduces protein expression of NLRP1 inflammasome and ameliorates spatial learning deficits in aged rats**. **(A) **Representative immunoblots of cleaved caspase-1, pannexin1 and P2X7R in hippocampal lysates of vehicle (Veh)-treated and probenecid (Pr)-treated 18-month-old rats. β-tubulin was used as an internal control. **(B) **Densitometric analysis of immunoblots from brain lysates of cleaved caspase-1 (Casp1), P2X7 receptor (P2X7R), and pannexin1 (PanX1). **(C-D) **Aged animals underwent behavioral testing following either probenecid or vehicle treatment. **(C) **In a hippocampal-dependent spatial learning task via Morris water maze, latency to platform was measured on days 1-3 and 8-10. Probenecid-treatment improved latency to platform measured on the final day of testing **(D) **Mean path length was determined on day 10 of testing and probenecid-treated rats demonstrated significantly shorter mean path lengths than vehicle-treated controls. Drug treatment was administered twice daily for 3 days (days 7-9). Data are presented as mean +/- SEM *p < 0.05, **p < 0.005 compared to vehicle. N = 6-8/per group.

Probenecid was previously shown to block inflammasome activity, specifically by inhibiting Pannexin 1 [15]. Here we show a significant decrease in cleaved caspase-1 (p = 0.0426, Figure 4B), P2X7 receptor (p = 0.0258, Figure 4B), and Pannexin1 (p = 0.004, Figure 4B) protein expression in the hippocampus following probenecid treatment in aged rats consistent with lower protein levels of IL-1β and IL-18. Treatment with probenecid also improved spatial learning performance in aged rats. Probenecid treated aged rats demonstrated lower latency to platform (p = 0.0287, Figure 4C) and mean path length (p = 0.015, Figure 4D) than vehicle-treated age-matched controls. These results demonstrate that treatment with probenecid reduces age-related increases in NLRP1 inflammasome activation in the aged hippocampus and ameliorates age-related cognitive deficits.

## Discussion

In this study we have shown for the first time an age-related heightened activation of the NLRP1 inflammasome system associated with increased inflammation and cognitive impairment in the hippocampus of aged rats. Our data show that aging promotes NLRP1 inflammasome activity resulting in processing of caspase-1 and upregulation of caspase-11. The neuronal NLRP1 inflammasome is a multiprotein complex consisting of inflammatory caspases-1 and -11, NLRP1, the adaptor protein ASC and the inhibitor of apoptosis protein XIAP. Assembly and activation of the NLRP1 inflammasome involves caspase -1 and -11 activation that subsequently leads to maturation and secretion of IL-1β and IL-18 [4-6]. Once secreted, these cytokines initiate inflammatory processes throughout the CNS. In addition, we have previously shown that the NLRP1 inflammasome interacts with the pore forming protein pannexin-1 and the purinergic receptor P2X7 [3-5]. Aging leads to significant cognitive impairment in hippocampal dependent spatial learning tasks [13] that may be associated with increased inflammatory cytokine production resulting from NLRP1 activation. Thus, the NLRP1 inflammasome constitutes an important arm of the innate CNS inflammatory response associated with aging in the hippocampus.

IL-1β is synthesized by neuronal and glial cells [16] and is released in response to injury, insult and stress [17-20]. We found that hippocampal neurons, which are generally not associated with immune functions, express NLRP1 inflammasome proteins. These results are in agreement with previous findings that show NLRP1 expression in neurons of the spinal [4] and cerebral cortex [5,6] after injury. Moreover, the cellular distribution of NLRP1 inflammasome proteins changed during the aging process. Our results suggest that NLRP1 inflammasome activity is fundamental for the processing of IL-1β and IL-18 and for the innate inflammatory response in aged neurons. However, ASC, caspase-1 and caspase-11 are also present in astrocytes, oligodendrocytes and microglial cells. Therefore, characterization of the composition and subcellular localization of inflammasomes in glia may provide a clearer insight into the mechanisms leading to cytokine secretion and cell death caused by caspase-1 with aging. Thus, aging-induced NLRP1 inflammasome activation in neurons could lead to reactive gliosis in neighboring cells mediated by IL-1 cytokines released from neurons.

The inflammasome in hippocampal neurons is a protein complex containing NLRP1 as a scaffolding protein that activates caspase-1 to promote IL-1β and IL-18 maturation associated with aging (see additional file 1 for immunoblotting results of IL1β and IL-18 in young and aged rats). Although the total levels of NLRP1 and ASC in lysates did not change significantly with aging, the proportions of other key components that form the NLRP1 inflammasome increased. These findings are in agreement with our previous work on NLRP1 inflammasome regulation following spinal cord [4] and traumatic brain injury [5] that showed similar changes in NLRP1 components after injury. Moreover, the inflammasome in normal tissues is speculated to be in an inactive state by binding to a putative inhibitor [21], but the nature of this inhibitor has not been identified. We previously suggested that XIAP in the NLRP1 inflammasome complex may inhibit caspase-1 activity preventing the activation and processing of IL-1β and IL-18 [3-5]. Moreover, aging-induced activation of the inflammasome was found to be associated with cleavage of XIAP into fragments. Cleavage of XIAP produces an N-terminal BIR1-2 fragment with reduced ability to inhibit caspases [22-24]. Therefore, aging-related XIAP cleavage may reduce the threshold for activation of caspase-1, leading to processing and secretion of IL-1β and IL-18.

Our results are in agreement with earlier studies that demonstrate systemic administration of IL-1β results in impaired hippocampal-dependent consolidation of memories in a fear-conditioning paradigm [25-27]. Neutralization of IL-1β blocked the deficits in hippocampal-dependent memory consolidation [25,28]. Other reports demonstrate that the aging process results in elevated concentrations of IL-1β at 15 months of age [29], whereas hippocampal IL-18, a closely related IL-1 proinflammatory cytokine increases in rats as early as 9 months of age [29]. Additionally, the age-specific elevation in these proinflammatory cytokines may influence deficits in long-term potentiation [30,31]. Further studies are needed to determine whether the NLRP1 inflammasome-induced activation and increased IL-1β and IL-18 observed in our study influence deficits in synaptic transmission leading to cognitive decline.

To date, microbial pathogen-associated molecules and toxins have been identified as key triggers of activation of inflammasomes [1,2]. However, recently, environmental [32-34] and neurodegenerative [35,36] stimuli have been identified that lead to IL-1β release by means of inflammasomes. With respect to the latter, researchers [36] demonstrated that the NLRP3 inflammasome is activated by fibrous particles of amyloid-beta that results in cleavage of caspase-1 and production of IL-1β in microglia and macrophages. However, it is not known whether amyloid-beta activates the NLRP1 inflammasome in neurons thus further enhancing production of IL-1β in the aging brain. Our recent work demonstrated that the pore-forming protein pannexin-1 in neurons and astrocytes transports the extracellular K^+ ^ions to stimulate the NLRP1 inflammasome in the cytoplasm after P2X7 receptors bind ATP [3]. Thus, it is possible that the aging-induced increases in pannexin-1 and P2X7 expression in hippocampal neurons observed in this study may facilitate K^+ ^influx, thereby initiating NLRP1 inflammasome activation.

Probenecid is an inhibitor of renal tubular transporters, regularly used in the treatment of gout. However, probenecid-sensitive transporters are found throughout the body including the immune privileged CNS [3,37-40]. Using *in vivo *microdialysis techniques, one study examined the transport of probenecid across the blood brain barrier (BBB), reporting the presence of probenecid in cerebrospinal fluid, brain interstitial fluid, and brain tissue tissue [41]. Therefore, in the present study, we chose a course of treatment based on the recent identification of probenecid as an inhibitor of inflammasome activation in neurons and astrocytes [3,15]. We were specifically interested in the effect of probenecid treatment in improving cognitive deficits observed in aged rats. Young rats did not demonstrate impairment in spatial learning and therefore were not tested in the probenecid study. We hypothesized that probenecid may reduce cognitive impairment in aged rats via inhibition of inflammasome activation in the hippocampus. Accordingly, our findings support the use of probenecid as an anti-inflammatory therapy in the CNS. The aging-induced elevation in inflammasome activation in the hippocampus was attenuated by probenecid treatment, resulting in improved cognitive outcomes for aged rats *in vivo*.

## Conclusions

Our main findings suggest that the aged brain experiences heightened activation of the NLRP1 inflammasome system with resultant increases in caspase 3 activation and inflammatory cytokines IL-1β and IL-18 corresponding to impaired spatial learning in aged rats. Since these cytokines contribute to the pathology of different neurodegenerative diseases such as Alzheimer disease [42,43] and Parkinson disease [44]. and are involved in a positive feedback mechanism in which more cytokines are produced upon cytokine release [45], it is reasonable to hypothesize that interfering with inflammasome activation may prove to be beneficial in delaying development of age-related neurodegenerative diseases in which the IL-1 inflammatory response plays a pathogenic role.

Additionally, our results suggest that inhibition of inflammasome activation in the aged brain, with systemic probenecid treatment, reduces caspase-1 activation and, in turn, reduces inflammatory cytokines, IL-1β and IL-18, which have been linked to age-related cognitive decline. Because probenecid is an inhibitor of protein transporters found throughout the body [3,37-40], it is possible that the effect of probenecid on cognitive performance may be multifactorial. Additionally, one must consider the potential exerted by probenecid to increase levels of neuroprotectants such as kynurenic acid (KYNA) when administered with the precursor of KYNA, L-kynurennine (L-KYN) in toxic disease models in rats [46-49]. The effect of probenecid on KYNA levels in the aged brain deserves further study. However, our data suggest that therapeutic intervention with probenecid alone or another agent that inhibits the NLRP1 inflammasome, with corresponding reduction in proinflammatory cytokines, may improve overall cognitive outcomes and reduce symptoms of normal cognitive aging. In addition, the therapeutic use of probenecid in young and aged animals with neuroinflammation due to disease or injury also deserves further study.

## Methods

### Animals

A total of 20 young (3 months, 220-250 grams) and 36 aged (18 months, 375-450 grams) male Fischer rats were acquired from Charles River Laboratories and the National Institute on Aging Colony respectively for this study. Animals were maintained on a 12:12 h (light: dark) cycle and given food *ad libitum*. All animals were housed according to National Institutes of Health and United States Department of Agriculture guidelines. The Institutional Animal Care and Use Committee of the University of Miami approved all animal procedures. Animals were submitted to behavioral testing or biochemical analysis following a 2 week acclimation period under standard laboratory conditions. For behavioral testing, n = 20/age group. For biochemical analyses, n = 6/age group.

### Probenecid treatment

Sixteen aged (18 month old) male Fischer 344 rats were randomized to receive probenecid (Alfa Aesar) or saline vehicle at a dose of 0.5 mg/kg by intraperitoneal (I.P.) injection twice daily for 3 days.

### Behavioral Testing

Spatial acquisition and retention was assessed using a Morris water maze navigational task [50]. A circular tank (122 cm diameter) in a room with visual cues was filled with water (21°C) made opaque with white paint. A platform (9.3 cm diameter) hidden just beneath the water surface was placed in the northeast quadrant. The path length (i.e., the distance traveled until locating the platform) and latency to find the platform were recorded and analyzed with Ethovision software (Noldus Information Technology). Latency and mean path length to platform was measured for days 1 - 3 of testing. Each day, the animals were given four trials and an average was determined. The animal was placed randomly at each of four starting points (north, south, east, and west) and allowed 60 s to find the hidden platform. After locating the platform, the animal remained on the platform for 10 s. If the animal did not locate the platform within 60 s, it was placed on the platform for 10 s. Following each trial, the animal was placed in a cage kept warm with an infrared heating lamp. Inter-trial interval was approximately 4 min. Testing days 1-3 for latency and day 3 for path length are reported for retention of the task. For the probenecid experiment, animals were tested on days 1 - 3 and 8 - 10. Probenecid or saline vehicle was administered on days 7 - 9. Behavioral testing was conducted similarly to the testing above with the placement of the hidden platform moved to the southwest quadrant for the second testing session. Latency to platform is reported for days 1-3 and 8-10. Mean path lengths are reported for day 10.

### Immunoblotting

Animals were anesthetized with 3% isoflurane/70% N_2_O/30% O_2 _for 5 minutes then sacrificed by decapitation. The bilateral hippocampi were dissected out at 4°C in saline and frozen in liquid nitrogen within 3 min of decapitation, and stored at -80 deg C. For detection of inflammasome components, receptors and inflammatory cytokines, a section of the right hippocampus of young and aged animals was homogenized in extraction buffer (20 mM Tris-HCl, pH: 7.5, 150 mM NaCl, 1% Triton X-100; 1 mM ethylenediaminetetraacetic acid, 1 mM ethylene glycol tetraacetic acid, 2.5 mM pyrophosphate, 1 mM -glycerophosphate) with protease and phosphatase inhibitors cocktail (Sigma). Proteins were resolved in 10-20% Tris-HCl Criterion precasted gels (Bio-Rad), transferred to polyvinylidene difluoride membranes (Applied Biosystems) and placed in blocking buffer (PBS, 0.1% Tween-20, 0.4% I-Block (Applied Biosystems) and then incubated for 1 h with: anti-IL-1β (1:1000, National Cancer Institute - 3ZD MAb), anti-IL-18 (1:1000, Abcam ab37640), anti-caspase-1 (1:1000, Imgenex - IMG5028), anti-caspase-11 (1:1000, Alexis Biochemicals, Axxora - ALX-210-818), anti-ASC (1:5000, Bethyl Laboratories, as described in de Rivero Vaccari et. al. 2008), anti-NLRP1 (1:1000, Bethyl Laboratories as described in de Rivero Vaccari et al., 2008), anti-pannexin-1 (1:1000, Invitrogen - 488100) and anti-P2X7 (1:1000, Alomone Labs - APR-004) followed by appropriate secondary horseradish peroxidase (HRP)-linked antibodies (Cell Signaling). Visualization of signal was enhanced by chemiluminescence using a phototope-HRP detection kit (Cell Signaling). To control for protein loading, immunoblots were stripped with Restore, Western blot stripping buffer (Pierce) and blotted for β-tubulin (1:5000, BD Biosciences Pharmingen). Quantification of band density was performed using UN-Scan-IT gel™ quantifying software (Silk Scientific), and data were normalized to β-tubulin. However IL-1β and IL-18 immunoblots were not quantified because these two proteins are secreted cytokines.

### Perfusion Fixation

Following behavior testing, young and aged animals were anesthetized with 3% isoflurane/70% N_2_O/30% O_2 _for 5 minutes and intracardially perfused with cold heparinized normal saline solution and subsequently with 4% paraformaldehyde solution. Brains were removed and placed in 4% paraformaldehyde at 4°C for 20 h. Then brains were placed in 20% sucrose in 0.1 M PBS and stored at 4° until sectioning through the hippocampus from 2.4 to 5.8 mm posterior to bregma. Sections were taken with a thickness of 50 μμm.

### Immunohistochemistry

Frozen sections were blocked by treatment with normal goat serum (Vector Laboratories), rinsed with 0.1 M phosphate-buffered saline (PBS; pH 7.4) and incubated overnight at 4°C with primary antibodies against the same proteins analyzed by immunoblotting at a dilution of 1:500. To determine the precise cellular distribution of caspase-1, pannexin-1 and P2X7, sections were double stained with the neuron specific marker mouse anti-neuronal nuclei (NeuN; Chemicon) and anti-caspase-1 (Upstate), anti-P2X7 (Alomone Labs) and anti-pannexin-1 (Invitrogen). Alexa-Fluor secondary antibody conjugates (Molecular Probes) were used as secondary antibodies. Controls using an irrelevant antibody of the same isotype were run in parallel to evaluate antibody specificity.

### Statistical Analysis

Data are expressed as mean +/- standard error (+/-SEM). Statistical comparisons between young and aged groups, and treatment groups were made using one-way analysis of variance, or repeated measures analysis of variance followed by Tukey's multiple comparison tests depending on the outcome measure. *P*-values of significance used were *p *< 0.05.

## Abbreviations

ASC: Apoptosis-associated-speck-like protein containing a caspase recruitment domain; BBB: Blood brain barrier; CNS: Central nervous system; IL: Interleukin; KYNA: kynurenic acid; L-KYN: L-kynurennine; LRR: leucine-rich repeat; NLR: nucleotide-binding domain, leucine-rich repeat containing receptor; NLRP1: Nucleotide-binding domain, leucine-rich repeat containing receptor protein 1; NLRP3: Nucleotide-binding domain, leucine-rich repeat containing receptor protein 3; SEM: standard error mean; XIAP: X-linked inhibitor of apoptosis.

## Competing interests

The authors declare that they have no competing interests.

## Authors' contributions

LM and JdR conceived of the study and experimental design. LM carried out behavioral testing, perfusion fixation, immunoblotting, and statistical analysis and drafted the manuscript. JdR carried out immunoblotting, immunohistochemistry and revised the manuscript. GD performed western blots. RK made significant contributions to the design of the study and helped to draft the manuscript. HB coordinated the study, provided intellectual feedback for the design of the study and participated in statistical analysis and data interpretation. All authors read and approved the final manuscript.

## Supplementary Material

Additional file 1 **Aging induces processing of IL-1β and IL-18 in the hippocampus**. Representative immunoblot analysis of hippocampal brain lysates of young (Y) and aged (A) animals. Brain lysates were immunoblotted with antibodies against IL-1β and IL-18. β-Tubulin was used as an internal standard and control for protein loading. Previous findings have shown that IL-1 cytokines in the brain are associated with the aging process. To establish whether aging activates these pro-inflammatory cytokines in the hippocampus, protein lysates from young and aged rats were analyzed for IL-1β and IL-18 by immunoblotting procedures. The levels of active, processed forms of IL-1β and IL-18 were higher in the aged animals than their younger counterparts, thus indicating that aging induces activation of these inflammatory cytokines in the hippocampus. Unlike IL-1α, IL-1β and IL-18 are cytokines that are active only after inflammasome processing. Therefore, since IL-1β and IL-18 are secreted cytokines quantification of these cytokines was not done for it would represent an inaccurate estimation due to the inability to determine the amount of IL-1 cytokines that are still in the cell and the amount that has been secreted. Instead, inflammasome activation was determined by measuring the protein levels of caspase-1 (See Figure 2). Click here for file
