# Supplementary figures and images for: Heightened inflammasome activation is linked to age-related cognitive impairment in Fischer 344 rats
Source: BMC Neurosci. 2011 Dec 1;12:123. doi: 10.1186/1471-2202-12-123 (PMC3259063; doi:10.1186/1471-2202-12-123)

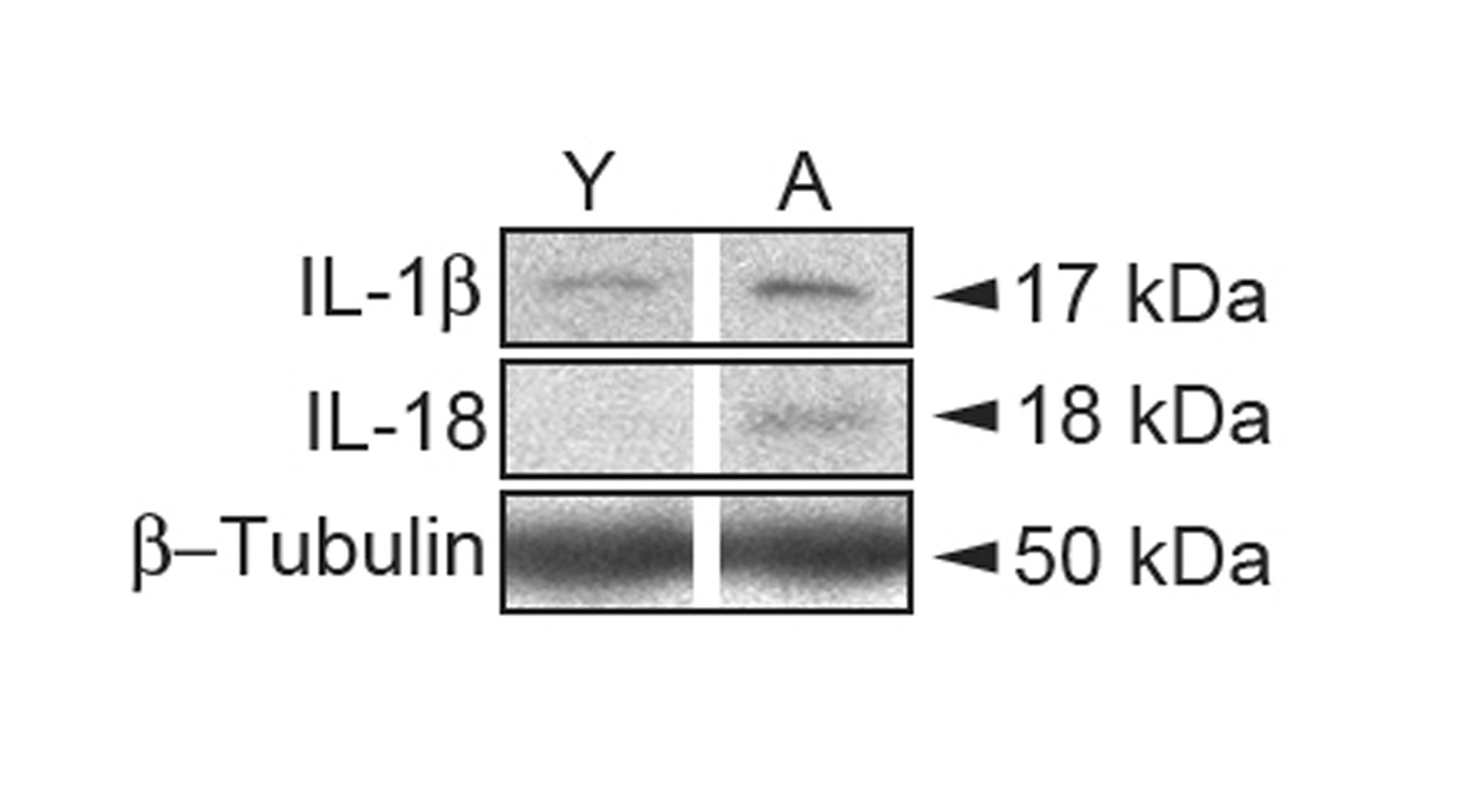

Supplement: Additional file 1 — Aging induces processing of IL-1β and IL-18 in the hippocampus. Representative immunoblot analysis of hippocampal brain lysates of young (Y) and aged (A) animals. Brain lysates were immunoblotted with antibodies against IL-1β and IL-18. β-Tubulin was used as an internal standard and control for protein loading. Previous findings have shown that IL-1 cytokines in the brain are associated with the aging process. To establish whether aging activates these pro-inflammatory cytokines in the hippocampus, protein lysates from young and aged rats were analyzed for IL-1β and IL-18 by immunoblotting procedures. The levels of active, processed forms of IL-1β and IL-18 were higher in the aged animals than their younger counterparts, thus indicating that aging induces activation of these inflammatory cytokines in the hippocampus. Unlike IL-1α, IL-1β and IL-18 are cytokines that are active only after inflammasome processing. Therefore, since IL-1β and IL-18 are secreted cytokines quantification of these cytokines was not done for it would represent an inaccurate estimation due to the inability to determine the amount of IL-1 cytokines that are still in the cell and the amount that has been secreted. Instead, inflammasome activation was determined by measuring the protein levels of caspase-1 (See Figure 2). [file 1471-2202-12-123-S1.JPEG]
